# Micro-scale, city-wide analysis of outdoor thermal comfort during heatwaves in high latitude cities: influence of building geometry and vegetation

**DOI:** 10.1007/s00484-025-03030-2

**Published:** 2025-09-22

**Authors:** Fredrik Lindberg, Nils Wallenberg, Sofia Thorsson, Marie Haeger-Eugensson, Jessika Lönn, Benjamin Holmberg, Martina Frid, Jesper Fahlström

**Affiliations:** 1 Department of Earth Science, University of Gothenburg, Medicinaregatan 7B, Gothenburg, Gothenburg, 413 90 Sweden; 2 COWI, Vikingsgatan 3, Gothenburg, 411 04 Sweden

**Keywords:** QGIS, Urban climate services, Heat wave mitigation

## Abstract

Urban citizens are particularly exposed to heat stress during heatwaves due to the urban climate conditions. Introducing more trees, changing building density and surface cover and materials are examples of planning measures that can be used to mitigate heat stress. One challenge as an urban planner is to have knowledge on which mitigation measure to implement to achieve the highest cooling effect with regards to outdoor heat stress at different spatial scales. The aim of this high-resolution modelling of outdoor thermal comfort on city-wide domains is to examine how different real-world urban settings reduce or exacerbate heat stress with regards to building density (plan area index), tree fraction, and ground cover. Here, we exploit the open-source tool Urban Multi-scale Environmental Predictor (UMEP), to investigate how real-world data on building density, tree fraction, and ground cover influence thermal comfort in the three largest cities in Sweden. Mean radiant temperature (T_mrt_) and two thermal comfort indices are calculated and compared: Physiological Equivalent Temperature (PET) and Universal Thermal Comfort Index (UTCI). Automated chain processes using Python scripting is demonstrated, making it possible to derive microscale outdoor thermal comfort information (2-meter resolution) using a standard personal computer and open data sources. Results show that tree fraction is the single most effective outdoor heat mitigation measure, especially in areas with low building density. Results also show that building fraction has a minor cooling effect. This is probably due to the fact that shadowing at street level is dominated by trees due their 3D characteristics including trunk zones. T_mrt_ shows very similar results compared with PET and UTCI, indicating that T_mrt_ can capture the spatial variations of heat stress during warm, clear and calm days. Since trees is the single most effective measure to mitigate heat stress, it should be incorporated when creating practical guidelines to resilient urban planning strategies against heat stress.

## Introduction

Heat stress risks are particularly pronounced in urban areas due to phenomena such as the nocturnal urban heat islands and uneven daytime exposure to outdoor heat, characterized by high radiant loads and reduced wind speeds (Oke et al. 2017). Radiant load, often quantified as the mean radiant temperature (T_mrt_), is a crucial meteorological parameter influencing human energy balance and thermal comfort during warm, clear weather (Mayer and Höppe 1987; Ali-Toudert and Mayer 2007). T_mrt_ is a key input in various thermal comfort indices, such as Physiological Equivalent Temperature (PET) (Höppe 1999) and Universal Thermal Climate Index (UTCI) (Fiala et al. 2012).

T_mrt_ results from the net effect of all shortwave and longwave radiation fluxes from the surroundings to which a human body is exposed. While intra-urban air temperature (T_air_) differences are relatively minor during the day, T_mrt_ can exhibit significant spatial variations over short distances (Emmanuel and Fernando 2007; Mayer et al. 2008; Lindberg and Grimmond 2011a; Thorsson et al. 2011). These microscale variations are primarily driven by shadow patterns created by obstructing objects like trees, buildings, and topography, as well as differences in the thermal and radiative properties of surrounding surface materials, such as albedo, emissivity, and heat capacity. On clear summer days with high irradiance, the highest T_mrt_ can be found near sunlit walls at noon or early afternoon (Lindberg et al. 2013), due to the high levels of direct and reflected shortwave radiation and emitted longwave radiation from sun-exposed surfaces. In these conditions, T_mrt_ can be significantly higher than T_air_. At night, when shortwave radiation is absent, T_mrt_ is nearly equal to T_air_.

Although T_mrt_ is the primary meteorological parameter contributing to spatial variations in daytime heat stress, thermal comfort indices (TC) like PET and UTCI also incorporate other external factors such as wind cooling effects, humidity, and ambient T_air_, which influence the energy balance and thermal comfort of the human body. While T_air_ and humidity show minimal spatial differences during the day, pedestrian wind speed can vary greatly in complex urban environments, potentially mitigating or exacerbating heat stress. Computational Fluid Dynamic models like ENVI-met (Simon 2016) and PALM4U (Maronga et al. 2020) can be used to estimate pedestrian wind speed. These models are computationally intensive and suitable for micro to local scale investigations. Therefore, when examining city-wide heat stress patterns, T_mrt_ is commonly used to describe spatial heat variations in larger urban areas (Jänicke et al. 2016; Lindberg et al. 2018; Buo et al. 2023). An exception is the pioneering study by Li et al. (2024), which calculated high-resolution UTCI maps for several major U.S. cities. However, wind patterns were calculated on a local rather than micro-scale, affecting the resulting high-resolution UTCI maps.

Understanding spatial variations in daytime heat stress in cities, both observational and modelling studies have shown that tall vegetation (e.g., trees and bushes) is highly effective in reducing heat by blocking of direct-beam solar radiation and lowering T_mrt_ (e.g., Bajsanski et al. 2016; Stojakovic et al. 2020) (e.g., Konarska et al., 2014; Lindberg et al. 2016a, b; Zhao et al. 2018; Abdi et al. 2020; Lee and Mayer 2021; HosseiniHaghighi et al. 2020). However, a comprehensive approach combining building density, ground cover, and tall vegetation has not yet been studied on a city-wide scale, where all major components (i.e., radiation and wind) are included.

The aim of this study is to examine how different real-world urban settings reduce or exacerbate heat stress related building density, tree cover and/or ground cover in high latitude cities. Thermal comfort indices of PET and UTCI, including high resolution variations of T_mrt_ and pedestrian wind speed is incorporated. Here, we exploit an open-source tool, the Urban Multi-scale Environmental Predictor (UMEP) (Lindberg et al. 2018), to investigate the influence of real-world information on building density, tree fraction and ground cover on thermal comfort (heat stress) in the three largest cities in Sweden; Stockholm, Göteborg and Malmö. Findings from this study give indications on the importance of different planning measures and how to mitigate heat stress in cities during episodes of extreme heat.

## Methods and materials

This study utilizes UMEP, an open-source climate service tool tailored for researchers and service providers such as architects, climatologists, energy experts, health professionals, and urban planners. UMEP offers a range of applications for outdoor thermal comfort, urban energy consumption, and climate change mitigation. It integrates advanced 1D and 2D models with tools that provide the necessary input data to operate these models (Lindberg et al. 2018). Spatial data from various scales and sources are accessed through QGIS, a cross-platform, free, open-source desktop geographic information system (GIS) application, which facilitates data viewing, editing, and analysis (QGIS Development Team 2025). The QGIS software system allows access to functionalities and GIS algorithms via Python scripting, enabling the setup of automated processes and workflows for large-scale processing, as demonstrated in this study.

### Data sources and study areas

The three largest cities in Sweden are included in this study: Stockholm, Gothenburg and Malmö. Table 1 presents some spatial characteristics of the three urban areas relevant for this study.

**Table 1:** Statistics on Spatial characteristics for the three cities used in this study

City	Spatial extent		Tall vegetation (< 2 magl)		Buildings	
*Stockholm* (59.3327° N, 18.0656° E)	km^2^ No. of grids (0.25 km^2^)	461.75 1847	Fraction Height (m) Volume (m^2^/m^3^)	0.37 11.18 4.31	Fraction Height (m) Volume (m^2^/m^3^)	0.10 8.45 1.27
*Gothenburg* (57.7089° N, 11.9746° E)	km^2^ No. of grids (0.25 km^2^)	181.0 724	Fraction Height (m) Volume (m^2^/m^3^)	0.31 9.99 3.37	Fraction Height (m) Volume (m^2^/m^3^)	0.13 8.42 1.43
*Malmö* 55.6050° N, 13.0038° E	km^2^ No. of grids (0.25 km^2^)	85.25 341	Fraction Height (m) Volume (m^2^/m^3^)	0.16 7.88 1.42	Fraction Height (m) Volume (m^2^/m^3^)	0.15 8.63 1.67

**Fig. 1:**
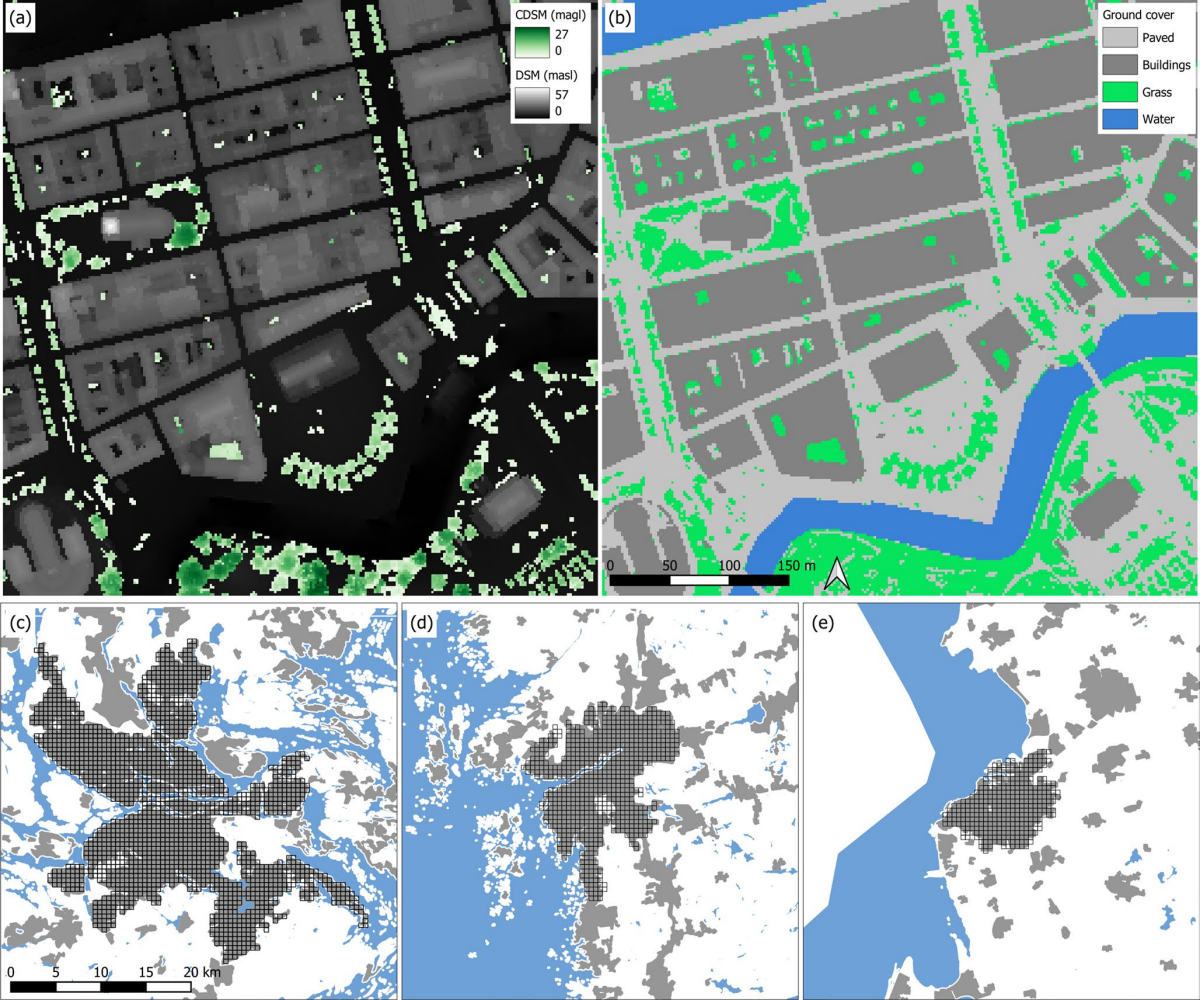
Example of the input geodata required for the modelling for a part of Gothenburg model domain (**a**) a ground and building digital surface model (DSM) overlaid by a vegetation canopy digital surface model (CDSM) and (**b**) ground cover. Pixel resolution is 2 m. Spatial extent of the three model domains for Stockholm (**c**), Gothenburg (**d**) and Malmö (**e**), respectively, indicated by the grids

Data required to analyse spatial indicators, and used as input in UMEP modelling, were derived from Light Detection and Ranging (LiDAR) screenings conducted by the Swedish Cadastral and Land Registration Authority (Lantmäteriet) from 2018 onwards. Following the method of Lindberg and Grimmond 2011b); Hedblom et al. (2017) and Lindberg et al. (2024), ground and building Digital Surface Models (DSM), vegetation Canopy Digital Surface Models (CDSM), and Land Covers (LC) were generated and gridded at 2-meter resolution from the LiDAR dataset. The code used for generating data can be accessed via a GitHub repository (Lindberg, 2025). Building footprint information is retrieved from Lantmäteriets Fastighetskarta (Swedish mapping, cadastral and land registration authority’s Property map). Figure 1 shows an example of the input geodata as well as the spatial extent of the urban areas for the three cities.

### Modelling framework

**Fig. 2:**
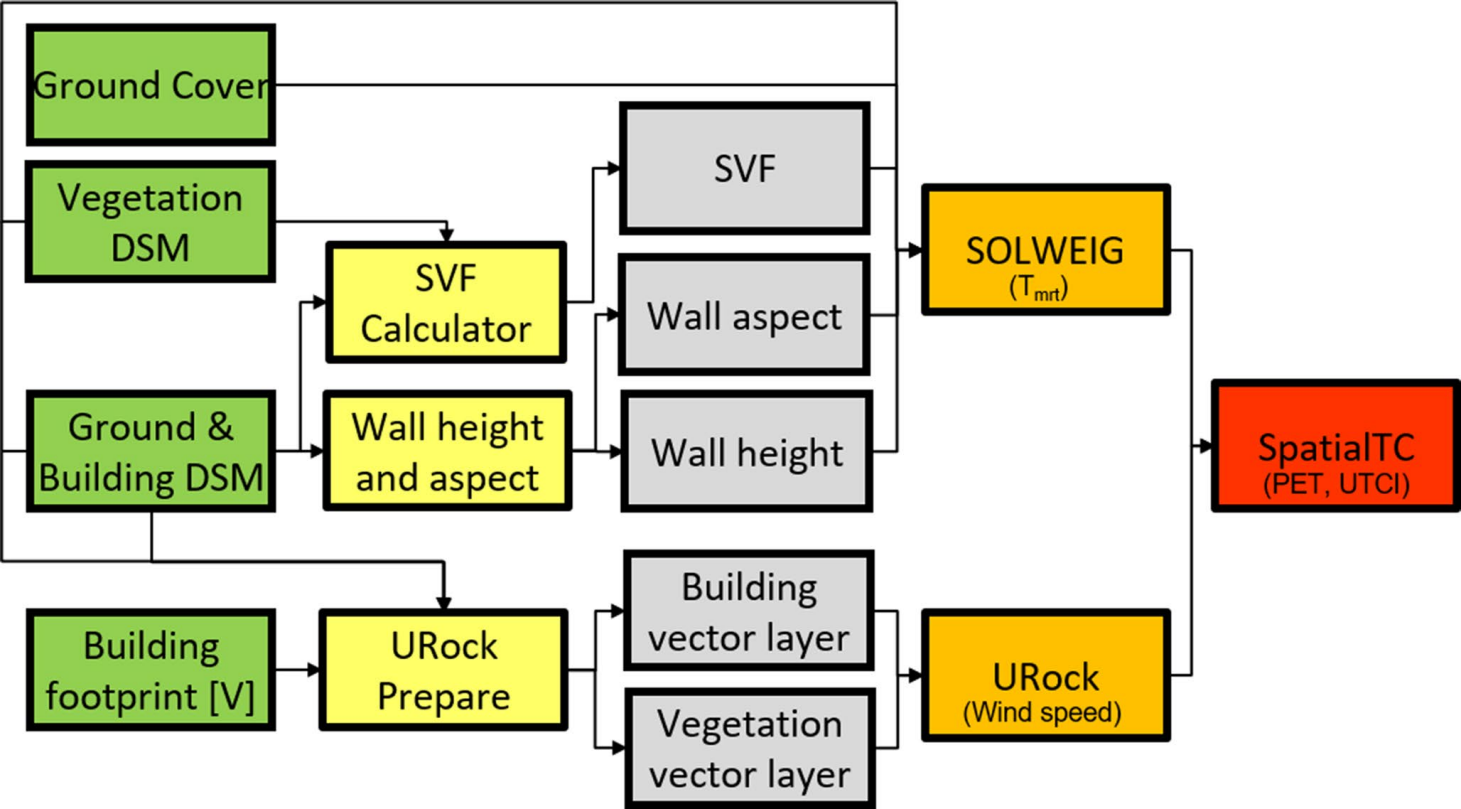
Workflow and geodata for analysing spatial variations of thermal comfort using UMEP; DEM – digital elevation model, DSM – digital surface mode. Green: Input geodata. Yellow: Pre-processor, Orange: Processor, Red: Post-processor, Grey: Intermediate geodata. (Tutorial available at: https://umep-docs.readthedocs.io/projects/tutorial/en/latest/Tutorials/SpatialTC.html#spatialtc)

Figure 2 shows the workflow implemented in UMEP to derive high resolution information on thermal comfort (i.e. PET and UTCI). The *SVF Calculator* calculates pixel-based sky view factors including both ground and buildings (DSM) as well as tall vegetation (CDSM) (Lindberg and Grimmond 2010; Wallenberg et al. 2023). Wall height and aspect are calculated by kernel filter processes explained in detail in Lindberg and Grimmond (2011), originally presented by Goodwin et al. (2009). Each grid was extended 100 m on each side (50 pixels) to reduce unwanted edge effects.

#### Meteorological forcing data

**Fig. 3:**
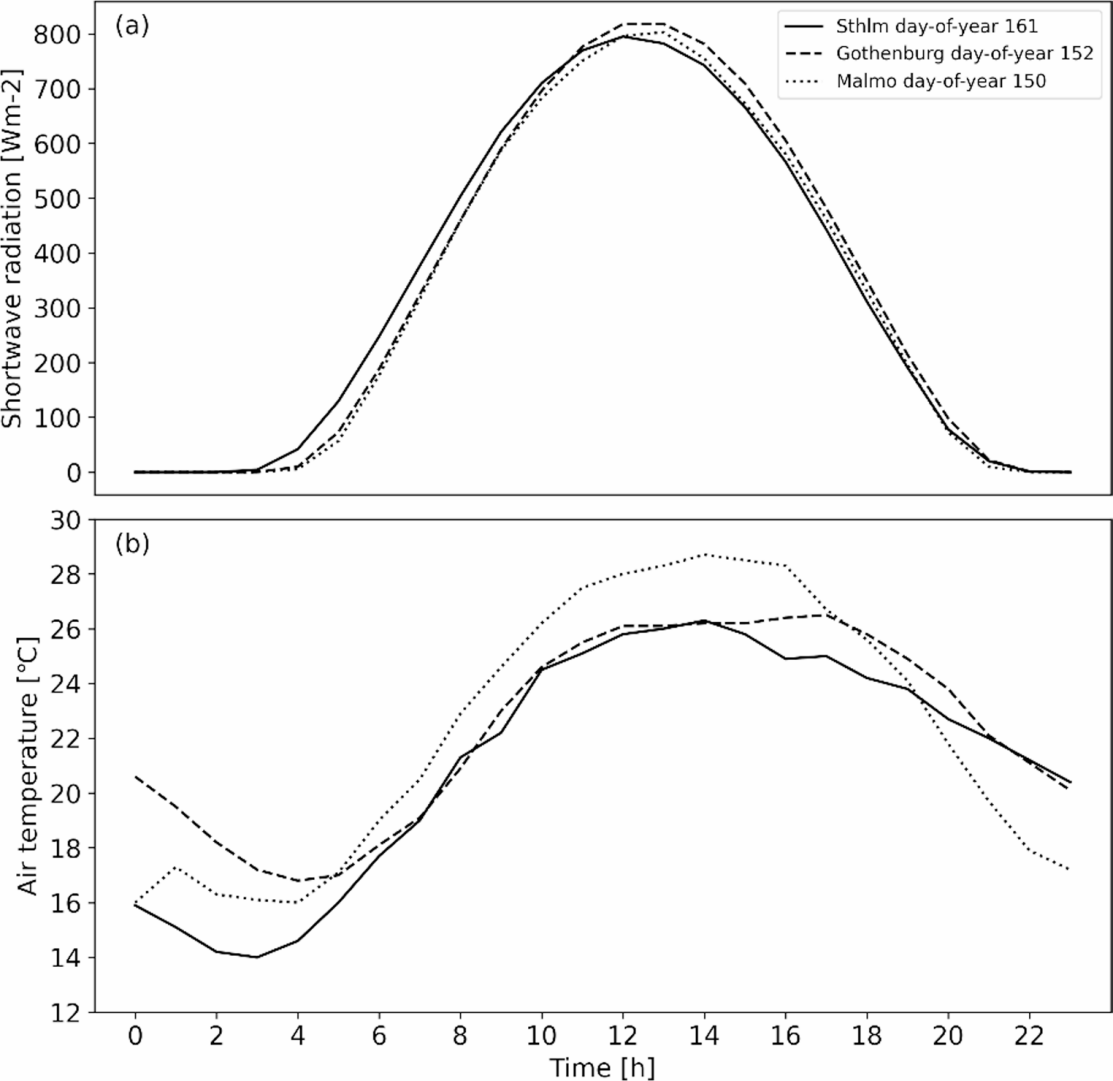
Global shortwave radiation (**a**) and air temperature (**b**) used as forcing meteorological data for Stockholm (solid), Gothenburg (dashed) and Malmö (dotted)

Similar meteorological forcing data for the three locations were selected based on Gower’s distance (Gower, 1971) for daily average global shortwave radiation and T_air_, and day of year. Days with high pressure situations, i.e., clear weather with low wind speed were selected. The day in Gothenburg, 2018-06-01, was used as the basis for selection of days in Stockholm and Malmö. Global shortwave radiation and T_air_ for the selected days are presented in Fig. 3. Wind speed and direction for each city (10 magl) is considered to be constant throughout the days of interest. For Stockholm, Gothenburg and Malmö, wind speed is set to 2 m/s representing an average wind speed for the days chosen i.e. a clear and calm high-pressure situation. Wind direction is set to 240°, 240° and 244°, respectively for each city, calculated as an average for the day of interest. The choice of input forcing data is further discussed in Sect. 3.3.

#### Radiation modelling

T_mrt_ was modelled using the SOLWEIG (SOlar LongWave Environmental Irradiance Geometry) v2022a, a microscale radiation model that simulates 3D radiation fluxes and T_mrt_ using commonly measured meteorological data (Lindberg et al. 2008, 2016a, b; Lindberg and Grimmond 2011a; Wallenberg et al. 2020, 2023). SOLWEIG requires the meteorological parameters of T_air_, relative humidity (RH), and global solar radiation, preferably partitioned into its diffuse and direct components, together with a DSM and a representative latitude/longitude for the location. The shadowing and view factor effects of trees and bushes were modelled with vegetation included as a separate DSM layer (Lindberg and Grimmond 2011a). Albedo and emissivity for buildings and vegetation were 0.20 and 0.95, respectively (Oke 1987). The transmissivity of shortwave and longwave radiation through vegetation was 3% and 0%, respectively (Konarska et al. 2013). T_mrt_ is calculated for a standing cylindrical person. Absorption coefficients for shortwave and longwave radiation were 0.7 and 0.97, respectively (Höppe 1992; VDI 1994). One warm and sunny day (hourly resolution during daytime) was modelled for each of the three cities (Fig. 3).

#### Pedestrian level wind modelling

Pedestrian level wind speed was simulated using URock v2023a, an open-source diagnostic model dedicated to wind field calculations in urban settings. URock is based on a quick method initially proposed by Röckle (1990). It is a two-step approach. In the first step, the wind speed and wind direction are initialized in several zones around wind obstacles based on a common wind profile representing wind field variations (speed and direction) with height. The location and size of the zones are derived from wind tunnel observations. The second step consists of balancing the airflow while minimizing the modifications of the initial wind field. For a detailed description of URock, see Bernard et al. (2023). In this study, the power law option is used to derive the general wind profile for the study area. The *p* exponent is adjusted based on urban geometry for each sub-grid (see Eq. 2 and Table 1 in Bernard et al. 2023). 3D wind vectors are derived at a 4-meter resolution, both vertically and horizontally then later re-gridded to 2 m to match the T_mrt_ maps created by SOLWEIG. Attenuation factor for vegetation is used based on larch plantation from Cionco (1972). Pedestrian wind speed derived at 1.5 m above ground level is used as input for further analysis (see next section). One wind speed and direction were used for the particular day modelled.

#### Spatial maps of thermal comfort

PET and UTCI are calculated using SpatialTC (Thermal Comfort), a tool in the post-processing section of UMEP that estimates pixel-based thermal comfort indices (PET, UTCI and COMFA (COMfort FormulA)). Both indices used require input meteorological variables of ambient T_air_, wind speed, humidity and radiation flux represented by T_mrt_. For PET human parameters are also needed. Standard values are used (age = 35 years, sex = male, weight = 75 kg, height 1.7 m, clo = 0.9 and activity = 80 W). Grid based output from URock (pedestrian wind speed) and SOLWEIG (T_mrt_) are used, together with input forcing data of T_air_ and RH.

## Results and discussions

### Influence of meteorological forcing and surface data on PET and UTCI

**Fig. 4:**
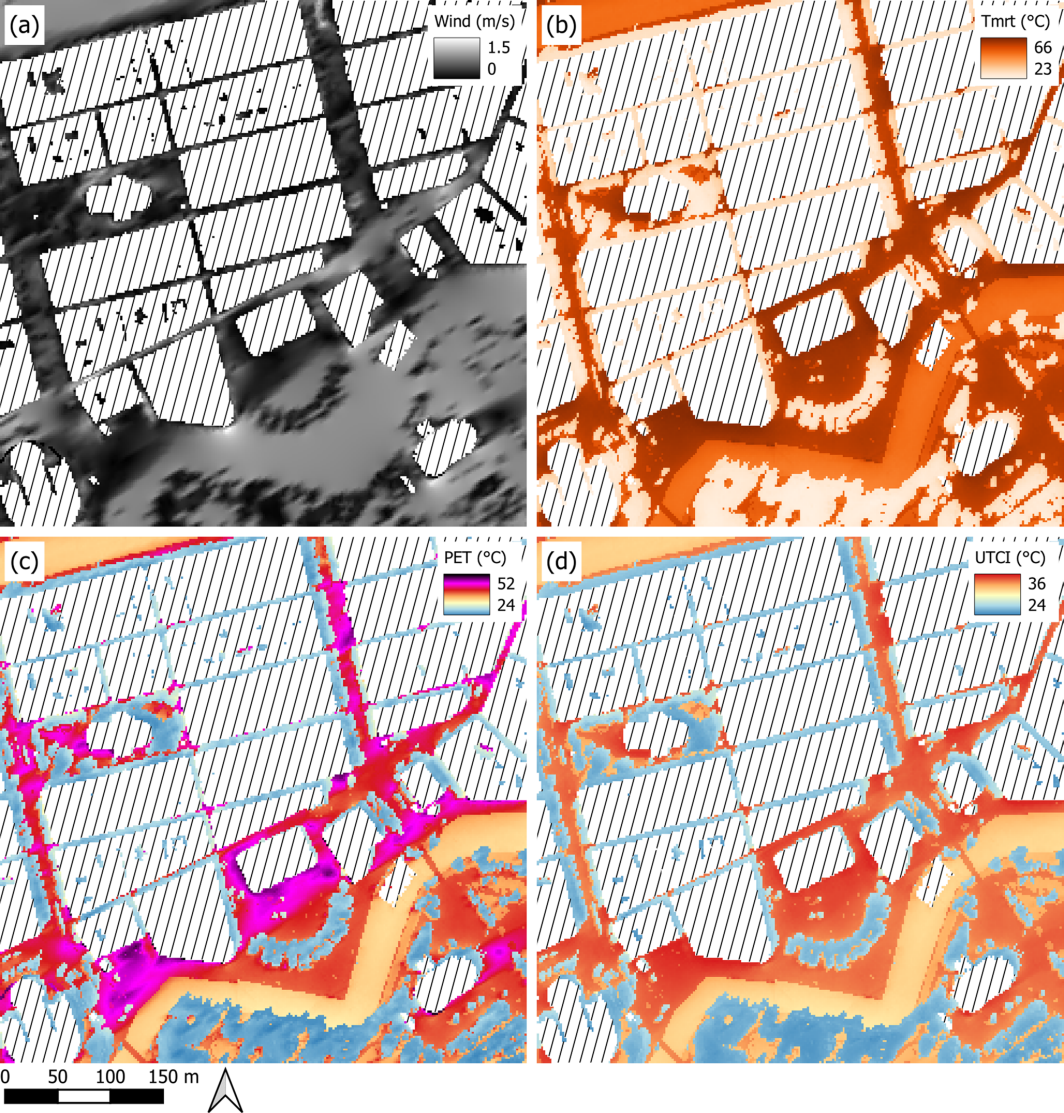
Example of the output form a part of Gothenburg model domain at 2 pm, 1 June 2018 (**a**) Pedestrian wind speed (1.5 m above ground level), (**b**) Mean radiant Temperature (T_mrt_), (**c**) Physiological Equivalent Temperature (PET) and (**d**) Universal Thermal Climate Index (UTCI). Pixel resolution is 2 m

Figure 4 shows an example of ground level (1.1 magl) output (wind fields, T_mrt_, PET and UTCI) for one sub-grid (500 × 500 m) in central Göteborg (2 pm, 2018, 1 June; 10 m wind speed 2 m/s, 2 m T_air_ is 26.2 °C, RH 37% and global shortwave radiation is 780 W/m^−2^). As shown, shadowing from buildings and high vegetation have a large influence on T_mrt_ as well as PET and UTCI. Variation in ground cover show a minor influence on the spatial variations of T_mrt_ and the two thermal indices. The influence of wind speed on PET and UTCI show that PET is much more sensitive for low wind speed compared to UTCI. Also, as UTCI implements a dynamic clothing model (Jendritzky et al. 2012), the large spatial differences seen in PET is not as evident in UTCI.

**Fig. 5:**
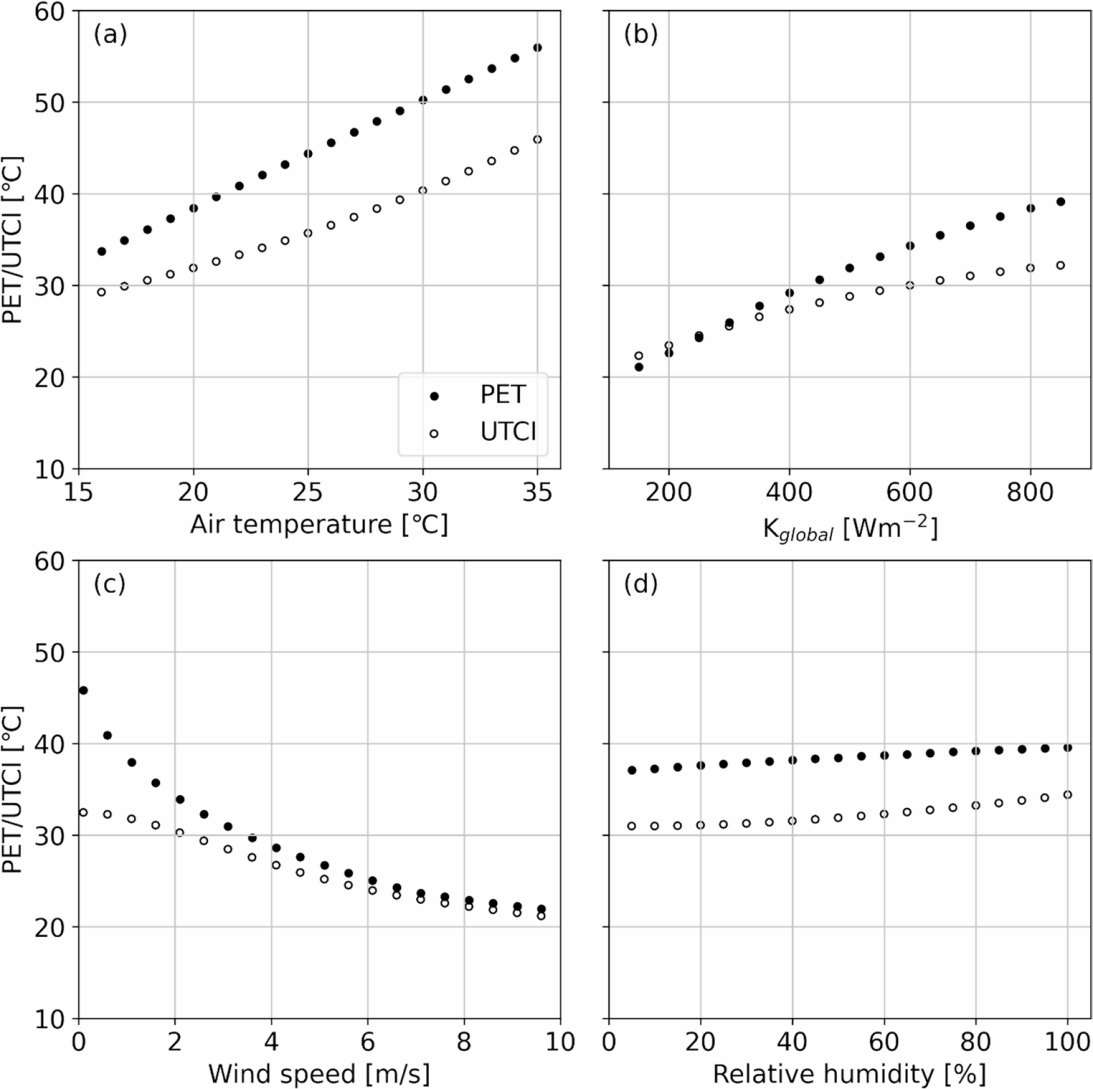
Scatter plots showing the resulting output of UTCI and PET for different inputs of (**a**) Tair (°C), (**b**) K_global_ (Wm^−2^), (**c**) wind speed (m/s), and (**d**) relative humidity (%) for a standard walking male. K_global_ is set to a fix value of 800 Wm^−2^ in a, c, and d; T_air_ to 20 °C in b–d; wind speed to 1.0 m/s in a, b, and d; and relative humidity to 50% in a–c. Calculations are for solar altitude and azimuth in Gothenburg, Sweden, 2025-06-30 14:00 LST

To further analyse how the meteorological forcing data (T_air_, global shortwave radiation, wind speed, and RH) influence PET and UTCI of a standard walking male in Gothenburg, Sweden, a sensitivity test was conducted as shown in Fig. 5. Only the higher end of the temperature span is examined (15–35 °C). The model by Reindl et al. (1990) was used in the sensitivity tests to partition direct and diffuse shortwave radiation from changes in incoming solar radiation. T_air_ (Fig. 5a) has relatively large and similar influence on both PET and UTCI throughout the temperature span investigated. Global radiation (> 400 Wm^−2^) has larger influence on PET compared to UTCI (Fig. 5b). Whereas PET show more of a linear trend, the influence of K_global_ on UTCI decreases as K_global_ increases. This could probably be explained due to fact that UTCI implements a dynamic clothing model (Fiala et al. 2012), whereas PET implements constant clothing values. Sensitivity of wind is very different when comparing the two indices, especially at low wind speed (< 2 m/s) (Fig. 5c). Again, UTCI is probably affected by the dynamic clothing model. PET tend to generate very high values (> 45 °C) when wind speed getting close to zero. Relative humidity (Fig. 5d) has a minor effect on either of the two indices. Also, RH and T_air_ does not vary considerably during daytime. Incoming global radiation and wind speed are two variables that can differ considerably on a spatial scale, where the differences in global radiation depend mainly on shadow patterns, which are considered in this study. Wind speed is influenced by the overall roughness as well as surrounding obstacle geometries, e.g., buildings and trees.

### City-wide analysis of T_mrt_, PET and UTCI

**Fig. 6:**
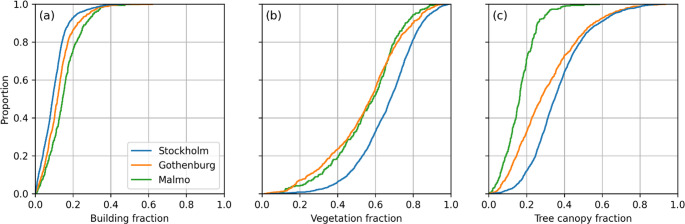
Cumulative frequency plots for (**a**) building fraction, (**b**) vegetation fraction and (**c**) tree canopy fraction for Stockholm, Gothenburg and Malmö

Cumulative distribution plots based on land cover for the three cities are shown in Fig. 6. Building fraction (building area/total area) is similar for the three cities, but Malmö is the densest followed by Gothenburg and Stockholm. Vegetation fraction (area low and high vegetation/total area) is similar for Göteborg and Malmö and somewhat higher for Stockholm. Regarding tree fraction (area tall vegetation/total area), Malmö stands out as having considerably lower tree fraction than Gothenburg and Stockholm.

**Fig. 7:**
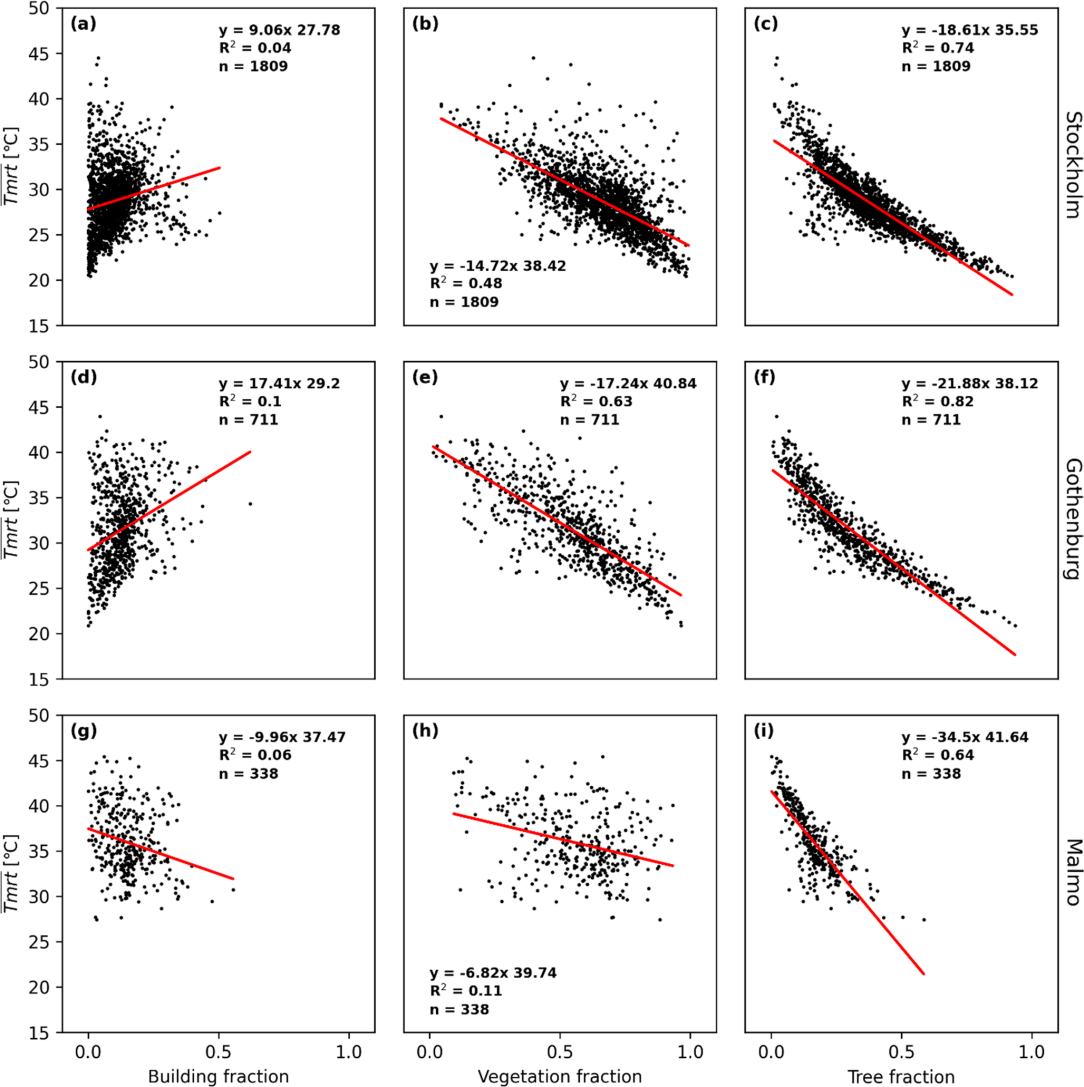
Scatterplots for average daytime T_mrt_ versus building fraction, vegetation fraction and tree canopy fraction for Stockholm, Gothenburg and Malmö. Each data point represents one 500 m x 500 m sub-grid domain at 2-meter pixel resolution

Scatterplots of average daytime T_mrt_ versus different measures of land cover fractions is shown in Fig. 7. Each data point represents one 500 m x 500 m sub-grid domain at 2-meter pixel resolution. Here, as shown previously, tree canopy fraction has the largest influence on average daytime T_mrt_. This has also been shown in previous studies (e.g. Lindberg et al. 2018; Lee and Mayer 2021). The relationship is non-linear, which has also been shown in previous studies (e.g. Lindberg et al. 2018; Klien 2022). This show that trees are the most effective heat mitigation measure at street level, especially in areas with no or few trees. Building fraction has almost no influence on daytime average T_mrt_. This is because building shadows is generated next to building footprints whereas as tall vegetation is a full 3D feature where shadows can occur underneath trees. Areas with high building fraction also reduce the potential area at street level where shadows from building objects can occur. This is not case for tall vegetation and hence, trees have a much stronger influence on daytime T_mrt_ at street level. There is also a clear pattern where high vegetation fraction result in low building fraction and vice versa (see Fig. 4 in Lönn et al. 2025). We also compare normalised building volumes with T_mrt_ and no improved correlation compared to building fraction was found (not shown).

**Fig. 8:**
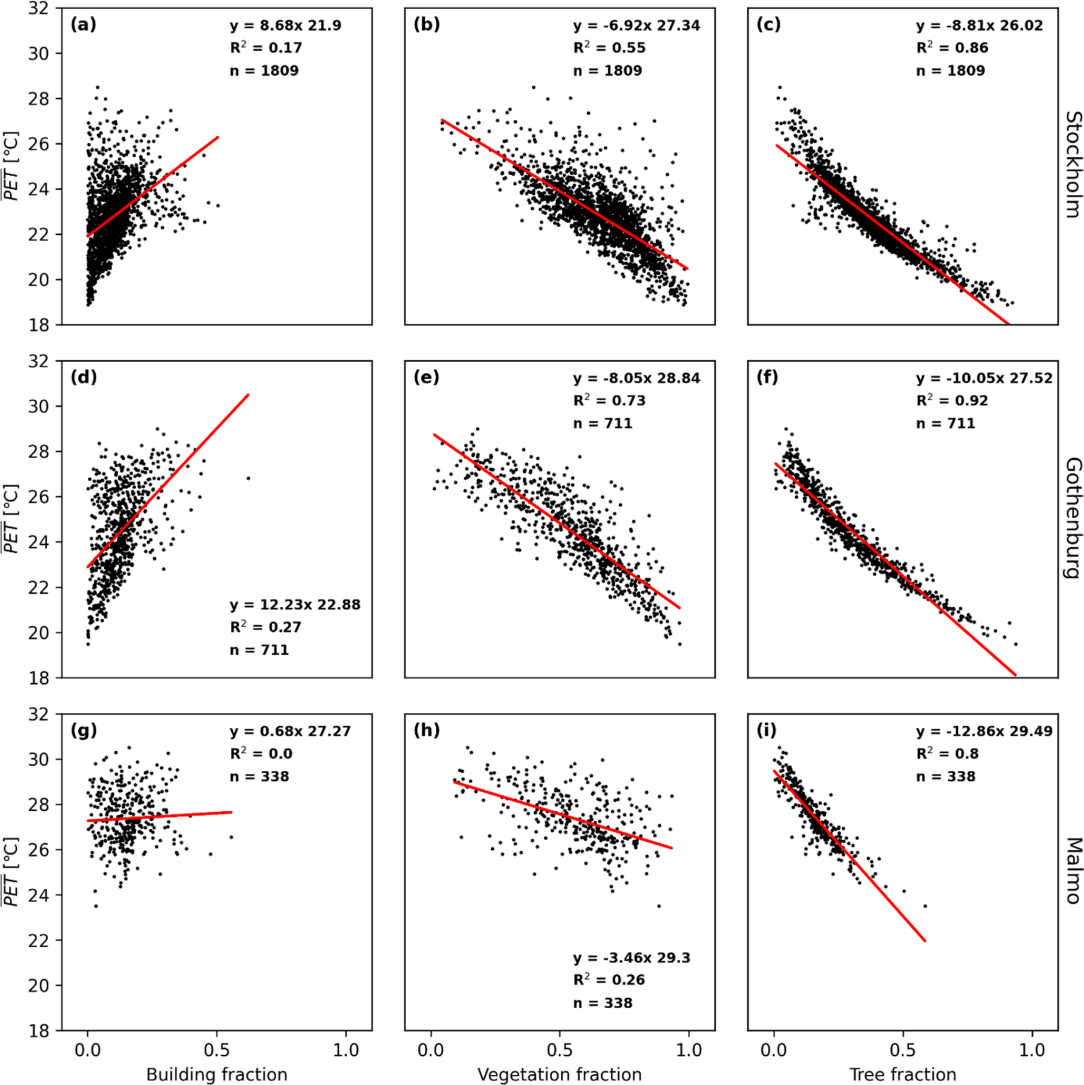
Scatterplots for average daytime physiological equivalent temperature (PET) versus building fraction, vegetation fraction and tree canopy fraction for Stockholm, Gothenburg and Malmö. Each data point represents one 500 m x 500 m sub-grid domain at 2-meter pixel resolution

**Fig. 9:**
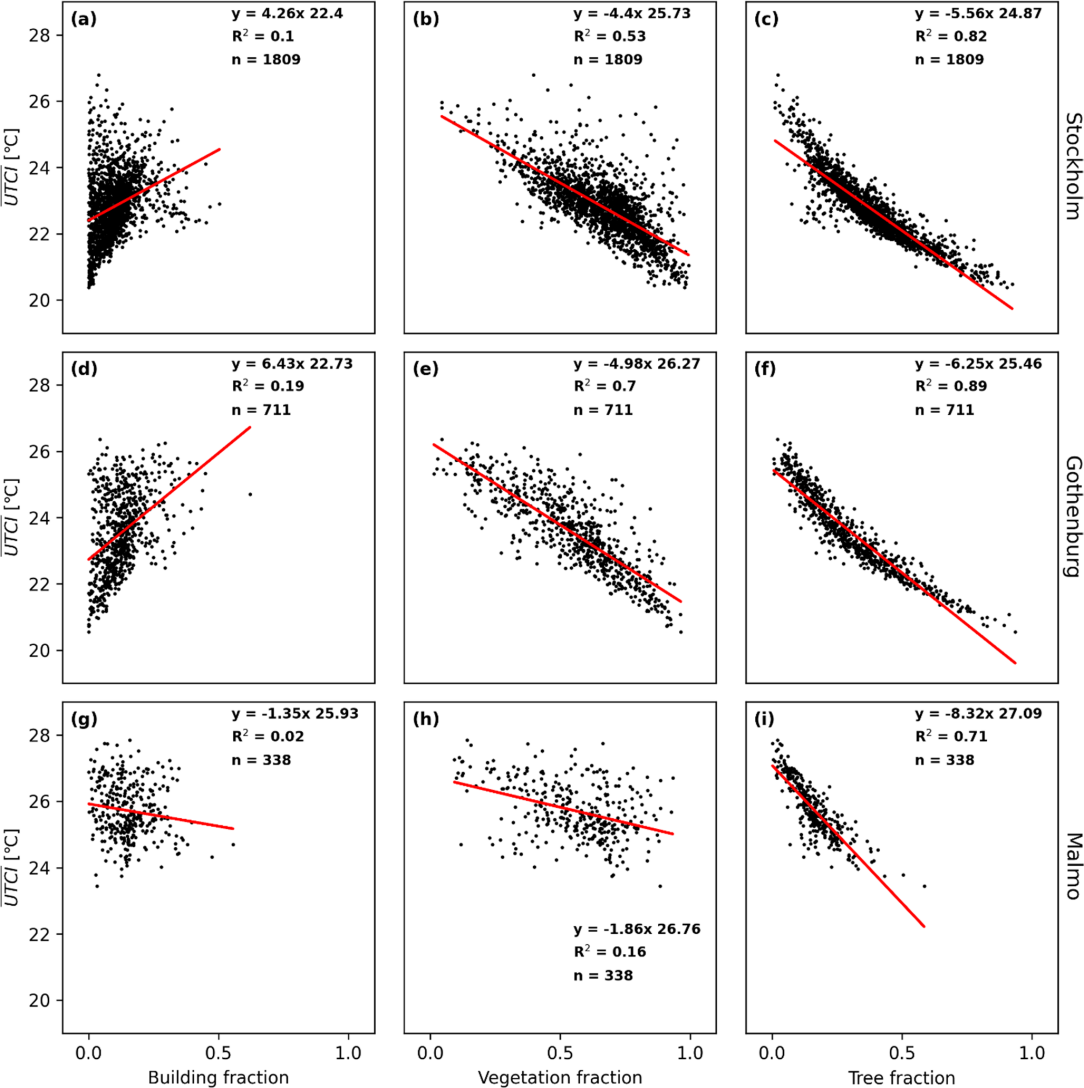
Scatterplots for average daytime universal thermal climate index (UTCI) versus building fraction, vegetation fraction and tree canopy fraction for Stockholm, Gothenburg and Malmö. Each data point represents one 500 m x 500 m sub-grid domain at 2-meter pixel resolution

The relationships are similar for PET (Fig. 8) and UTCI (Fig. 9), demonstrating that T_mrt_ is the most important meteorological variable that influences outdoor thermal comfort during warm and calm weather. The correlation between building fraction and daytime average T_mrt_ is somewhat higher for PET and UTCI compared to T_mrt_ indicating that pedestrian wind is influenced by buildings and thus influences the two indices. However, the correlation for building fraction is still low compared to both total vegetation fraction and tree fraction for all three cities.

### Limitations and uncertainties

This study demonstrates the importance of spatial characteristics on outdoor thermal comfort and can be used to examine what measures that influence T_mrt_, PET and UTCI on a city-wide scale. However, there are several limitations and uncertainties that should be discussed when interpreting the results and drawing conclusions from this kind of simulation study. The model simulations are conditional on the urban setting used, which in a more parametric case has a clear directionality with streets and buildings aligned e.g. along northwest– southeast and southwest – northeast axes. This directionality could accentuate the view that southwest-facing walls are prone to potential heat stress (e.g. Lindberg et al. 2016a, b). This study aims to minimize this effect by using real-world data for whole cities trying to capture the variations that can be found within a city.

The meteorological forcing used is very similar between the three domains but not equal. This could have a minor effect on the results. However, this study aims to represent real world examples of hot days for three cities which is accomplished through the method presented in Sect. 2.2.1. This study only examines daytime situations where the microscale radiation and wind filed patterns affect thermal comfort. Further studies should also include nocturnal situations where the local scale T_air_ is the main driver for heat related issues in urban areas related to the nocturnal urban heat island.

Potentially, pedestrian wind field could have been calculated for each time step. Here, only one speed and direction are used which could have implications. Nevertheless, wind speed and direction for the three days chosen were relatively constant and should not have been so different if new wind fields were generated for each timestep. This also makes it easier to compare the three cities. As shown in Fig. 5, both PET and UTCI is somewhat sensitive to T_air_ but not to RH. T_air_ are not spatially varying over the domains but since only daytime occasions are considered, T_air_ should not vary considerably in space.

The LiDAR data used to create all geodata for this study is of very high accuracy and precision. With that said, generating surface models from LiDAR point clouds could also include unwanted objects such as lifting cranes, containers etc. However, the overall results are of very high quality.

The models and algorithms used in this study have all been executed on single personal computer (Inter Core i5-9500 @ 3.0 GHz, 32 Gb RAM) resulting in long computation time (weeks). This could have been optimised in many ways, the most evident, distributing calculations on to graphical processing units (GPU). Li and Wang (2021) have translated parts on the workflow, such as sky view factor calculations and radiation modelling, showing a decrease in computation by 99% compared to regular central processing unit (CPU) computation. Furthermore, calculation could have also been distributed onto a high-performance computing (HPC) system which would have decreased computation time considerably through parallelisation.

This study is geographically constrained to the Nordic region and the cities examined in this study. However, looking at the complete built-up area of the three cities, a large variation in vegetation cover and building density is considered. This makes it possible to provide findings and give recommendations on how vegetation and buildings affect daytime heat stress also outside of the Nordic region. The most noticeable difference looking at other regions would foremost be sun positions, which will affect the amount of solar radiation reaching the ground floor in the urban environment. In such environment, tall vegetation would have an even more prominent effect on heat stress mitigation since trees in shadow of buildings would potentially be reduced. A future study employing the same modelling workflow could include other geographical and urban settings as exemplified by Thorsson et al. (2017) where it is clearly shown that regional climate has a large effect on radiant heat loads in urban areas across Europe.

## Concluding remarks

Urban citizens are particularly exposed to heat stress during heatwaves due to the urban climate conditions. Introducing more trees, altering building density, and changing ground cover materials are examples of planning measures that can be used to mitigate heat stress. Changing tree fraction and ground cover is relatively easy compared to altering building fraction which is a more of a constant over time. One challenge as an urban planner, is to have knowledge on which mitigation measure to implement to achieve the highest cooling effect. By exploiting open data and open-source tools, our results complement other studies and show that tree fraction is the single most effective heat mitigation factor, especially in areas with low building fraction. We also show that building fraction has a minor cooling effect related to T_mrt_. T_mrt_ show very similar result compared with PET and UTCI, indicating that T_mrt_ can capture the spatial patterns of heat stress during warm, clear and calm days. The correlation is slightly higher when the comfort indices PET and UTCI is used. This indicate that buildings increase roughness and decrease pedestrian wind speed, which could lower heat stress due to convective cooling from the human body. Even though wind has a subordinate influence on thermal comfort during weather situations causing heat stress (clear, warm and calm), it can, to some extent, reduce (or exacerbate) heat stress in outdoor urban settings. The sensitivity analysis indicates that the two thermal indices, PET and UTCI, respond differently to external meteorological factors. Both indices are equally influenced by variations in air temperature (T_air_) and relative humidity (RH). While T_air_ has a significant impact, variations in RH show almost no influence on either PET or UTCI. Regarding wind speed, PET is highly sensitive to low wind speeds, which can result in very high PET values when wind speed is close to zero. In contrast, UTCI does not exhibit the same pattern, likely due to its inclusion of a dynamic clothing model. Similar trends are observed with global radiation (K_global)_, where increasing values have a greater effect on PET compared to UTCI.

Findings show that trees is the most effective measure to mitigate heat stress and should be incorporated when creating practical guidelines to resilient urban planning strategies against heat stress. Increasing low vegetation in general have less of an effect for daytime heat mitigation than trees, due to the lack of shading created from grass and low bushes. In general, vegetation can also have other positive effects such as reducing nocturnal urban heat islands, increasing passive and active recreation, biodiversity and storm water management in urban areas.

## Data Availability

The datasets and code generated during and/or analysed during the current study are available from the corresponding author on reasonable request.

## References

[CR1] Abdi B, Hami A, Zarehaghi D (2020) Impact of small-scale tree planting patterns on outdoor cooling and thermal comfort. Sustain Cities Soc 56:102085. 10.1016/j.scs.2020.102085

[CR2] Ali-Toudert F, Mayer H (2007) Effects of asymmetry, galleries, overhanging façades and vegetation on thermal comfort in urban street canyons. Solar Energy 81:742–754

[CR3] Bajsanski I, Stojakovic V, Jovanovic M (2016) Effect of tree location on mitigating parking lot insolation. Comput Environ Urban Syst 56:59–67. 10.1016/j.compenvurbsys.2015.11.006

[CR4] Bernard J, Lindberg F, Oswald S (2023) URock 2023a: an open-source GIS-based wind model for complex urban settings. Geosci Model Dev 16:5703–5727. 10.5194/gmd-16-5703-2023

[CR5] Buo I, Sagris V, Jaagus J, Middel A (2023) High-resolution thermal exposure and shade maps for cool corridor planning. Sustain Cities Soc 93. 10.1016/j.scs.2023.104499

[CR6] Cionco RM (1972) A wind-profile index for canopy flow. Bound-Lay Meteorol 3:255–263. 10.1007/BF02033923

[CR7] Emmanuel R, Fernando HJS (2007) Urban heat islands in humid and arid climates: role of urban form and thermal properties in Colombo, Sri Lanka and Phoenix, USA. Clim Res 34:241–251

[CR8] Fiala D, Havenith G, Bröde P, Kampmann B, Jendritzky G (2012) UTCI-fiala multi-node model of human heat transfer and temperature regulation. Int J Biometeorol 56(3):429–441. 10.1007/s00484-011-0424-7 21503622 10.1007/s00484-011-0424-7

[CR9] Goodwin NR, Coops NC, Tooke TR, Christen A, Voogt JA (2009) Characterizing urban surface cover and structure with airborne lidar technology. Can J Remote Sens 35:297–309

[CR48] Gower, J. C. (1971). A General Coefficient of Similarity and Some of Its Properties. Biometrics, 27(4), 857–871. 10.2307/2528823

[CR10] Hedblom M, Lindberg F, Vogel E, Wissman J, Ahrné K (2017) Estimating urban lawn cover in space and time: case studies in three Swedish cities. Urban Ecosyst 20(5):1109–1119. 10.1007/s11252-017-0658-1

[CR13] HosseiniHaghighi S, Izadi F, Padsala R, Eicker U (2020) Using climate-sensitive 3D city modeling to analyze outdoor thermal comfort in urban areas. ISPRS Int J Geo-Inf 9(11):688. 10.3390/ijgi9110688

[CR11] Höppe P (1992) A new procedure to determine the mean radiant temperature outdoors. Wetter Leben 44:147–151

[CR12] Höppe P (1999) The physiological equivalent temperature – a universal index for the biometeorological assessment of the thermal environment. Int J Biometeorol 43:71–75. 10.1007/s004840050118 10552310 10.1007/s004840050118

[CR15] Jendritzky G, de Dear R, Havenith G (2012) UTCI — why another thermal index? Int J Biometeorol 56:421–428 22187087 10.1007/s00484-011-0513-7

[CR14] Jänicke B, Meier F, Lindberg F, Schubert S, Scherer Dieter (2016) Towards city-wide, building-resolving analysis of mean radiant temperature. Urban Climate 15:83–98

[CR16] Klien R (2022) Access, to urban cool and green spaces for old, adults in gothenburg – a gis-based analysis of radiant heat. Degree project for master of science (120 hec). Department of earth sciences, University of Gothenburg, Sweden. https://hdl.handle.net/2077/74598

[CR17] Konarska J, Lindberg F, Larsson A, Thorsson S, Holmer B (2013) Transmissivity of solar radiation through crowns of single urban trees—application for outdoor thermal comfort modelling. Theoret Appl Climatol 117:363–376

[CR18] Lee H, Mayer H (2021) Solar elevation impact on the heat stress mitigation of pedestrians on tree-lined sidewalks of EW street canyons – analysis under central European heat wave conditions, urban forest. Urban Green 58:126905. 10.1016/j.ufug.2020.126905

[CR21] Lindberg F (202 5 ) LidartoDSMs. GitHub. https://github.com/biglimp/LidarToDSMs. Accessed 6 Feb 2025

[CR22] Lindberg F, Grimmond C (2010) Continuous sky view factor from high resolution urban digital elevation models. Clim Res 42(3):177–183

[CR23] Lindberg F, Grimmond C (2011a) The influence of vegetation and Building morphology on shadow patterns and mean radiant temperature in urban areas: model development and evaluation. Theoret Appl Climatol 105(3):311–323

[CR24] Lindberg F, Grimmond CSB (2011b) Nature of vegetation and building morphology characteristics across a city: influence on shadow patterns and mean radiant temperatures in London. Urban Ecosyst 14:617–634

[CR29] Lindberg F, Grimmond CSB, Gabey A, Huang B, Kent CW, Sun T, Theeuwes NE, Järvi L, Ward HC, Capel-Timms I, Chang Y, Jonsson P, Krave N, Liu D, Meyer D, Olofson KFG, Tan J, Wästberg D, Xue L, Zhang Z (2018) Urban multi-scale environmental predictor (UMEP): an integrated tool for city-based climate services. Environ Model Softw 99:70–87

[CR25] Lindberg F, Holmer B, Thorsson S (2008) SOLWEIG 1.0 – modelling spatial variations of 3D radiant fluxes and mean radiant temperature in complex urban settings. Int J Biometeorol 52:697–713 18523814 10.1007/s00484-008-0162-7

[CR26] Lindberg F, Holmer B, Thorsson S, Rayner D (2013) Characteristics of the mean radiant temperature in high latitude cities—implications for sensitive climate planning applications. Int J Biometeorol 58(5):613–627 23456372 10.1007/s00484-013-0638-y

[CR28] Lindberg F, Lau K, Rayner D, Thorsson S (2016b) The impact of urban planning strategies on heat stress in a climate-change perspective. Sustain Cities Soc 25:1–12

[CR47] Lindberg F, Lindström A, Stålnacke V, Thorsson S, Destouni G (2024) Observations and modelling of mosquito prevalence within urban areas – A case study from Uppsala, Sweden. Urban Ecosystems. 27:1191-1205 10.1007/s11252-024-01511-7

[CR27] Lindberg F, Onomura S, Grimmond CSB (2016a) Influence of ground surface characteristics on the mean radiant temperature in urban areas. Int J Biometeorol 60(9):1439–1452 26852384 10.1007/s00484-016-1135-x

[CR19] Li X, Wang G (2021) GPU parallel computing for mapping urban outdoor heat exposure. Theor Appl Climatol. 10.1007/s00704-021-03692-z

[CR20] Li X, Wang G, Zaitchik B, Hsu A, Chakraborty TC (2024) Sensitivity and vulnerability to summer heat extremes in major cities of the United States. Environ Res Lett 19:9. 10.1088/1748-9326/ad6c64

[CR30] Lönn J, Åström C, Lindberg F, Thorsson S (2025) Heat related mortality among older adults in Sweden - the influence of tree canopy coverage and Building fraction. The Lancet, Planetary Health. In review

[CR31] Maronga B, Banzhaf S, Burmeister C, Esch T, Forkel R, Fröhlich D, Fuka V, Gehrke KF, Geletič J, Giersch S, Gronemeier T, Groß G, Heldens W, Hellsten A, Hoffmann F, Inagaki A, Kadasch E, Kanani-Sühring F, Ketelsen K, Khan BA, Knigge C, Knoop H, Krč P, Kurppa M, Maamari H, Matzarakis A, Mauder M, Pallasch M, Pavlik D, Pfafferott J, Resler J, Rissmann S, Russo E, Salim M, Schrempf M, Schwenkel J, Seckmeyer G, Schubert S, Sühring M, von Tils R, Vollmer L, Ward S, Witha B, Wurps H, Zeidler J, Raasch S (2020) Overview of the PALM model system 6.0. Geosci Model Dev 13:1335–1372. 10.5194/gmd-13-1335-2020

[CR33] Mayer H, Holst J, Dostal P, Imbery F, Schindler D (2008) Human thermal comfort in summer within an urban street canyon in central Europe. Meteorol Z 17:241–250

[CR32] Mayer H, Höppe P (1987) Thermal comfort of man in different urban environments. Theor Appl Climatol 38:43–49

[CR34] Oke T (1987) Boundary layer climates. Routledge, Cambridge, p 435

[CR35] Oke TR, Mills G, Christen A, Voogt JA (2017) Urban climates. Cambridge University Press

[CR36] QGIS Development Team (2025) QGIS geographic information system. Open-source geospatial foundation project. http://qgis.osgeo.org

[CR38] Röckle R (1990) Bestimmung der Strömungsverhältnisse im Bereich komplexer Bebauungsstrukturen, PhD thesis, 46116929

[CR37] Reindl DT, Beckman WA, Duffie JA (1990) Diffuse fraction correlations. Sol Energy 45(1):1–7. 10.1016/0038-092X

[CR39] Simon H (2016) Modeling urban microclimate: development, implementation and evaluation of new and improved calculation methods for the urban microclimate model ENVI-met, Dissertation zur Erlangung des Grades Doktor der Naturwissenschaften im Promotionsfach Geographie am Fachbereich Chemie, Pharmazie und Geowissenschaften der Johannes Gutenberg-Universität Mainz, Mainz

[CR40] Stojakovic V, Bajsanski I, Savic S, Milosevic D, Tepavcevic B (2020) The influence of changing location of trees in urban green spaces on insolation mitigation, urban forest. Urban Green 126721 10.1016/j.ufug.2020.126721

[CR41] Thorsson S, Lindberg F, Bjorklund J, Holmer B, Rayner D (2011) Potential changes in outdoor thermal comfort conditions in Gothenburg, Sweden due to climate change: the influence of urban geometry. Int J Climatol 31:324–335

[CR42] Thorsson S, Rayner D, Lindberg F, Monteiro A, Katzschner L, Lau K, Campe S, Katzschner A, Konarska J, Onomura S, Velho S, Holmer B (2017) Present and projected future mean radiant temperature for three European cities. Int J Biometeorol 61:1531–1543. 10.1007/s00484-017-1332-2 28447175 10.1007/s00484-017-1332-2 PMC5599478

[CR43] VDI (1994) Environmental meteorology, interactions between atmosphere and surface; calculation of short-and long wave radiation. Part I: Climate VDI 3789, part 2: VDI/DIN- handbuch reinhaltung der luft. Band 1b, Düsseldorf

[CR45] Wallenberg N, Lindberg F, Holmer B, Rayner D (2023) An anisotropic parameterization scheme for longwave irradiance and its impact on radiant load in urban outdoor settings. Int J Biometeorol. 10.1007/s00484-023-02441-3 10.1007/s00484-023-02441-3 PMC10070231 36826592

[CR44] Wallenberg N, Lindberg F, Holmer B, Thorsson S (2020) The influence of anisotropic diffuse shortwave radiation on mean radiant temperature in outdoor urban environments. Urban Clim 31(2020). 10.1016/j.uclim.2020.100589

[CR46] Zhao Q, Sailor DJ, Wentz EA (2018) Impact of tree locations and arrangements on outdoor microclimates and human thermal comfort in an urban residential environment, urban forest. Urban Green 32:81–91. 10.1016/j.ufug.2018.03.022

